# Dengue fever mapping in Bangladesh: A spatial modeling approach

**DOI:** 10.1002/hsr2.2154

**Published:** 2024-05-27

**Authors:** Indrani Sarker, Md. Rezaul Karim, Sefat E‐Barket, Mehedi Hasan

**Affiliations:** ^1^ Department of Statistics and Data Science Jahangirnagar University Dhaka Bangladesh

**Keywords:** Bayesian hierarchical framework, BYM2, Conditional Autoregressive model, convolution model, dengue, spatial modeling

## Abstract

**Background:**

Epidemics of the dengue virus can trigger widespread morbidity and mortality along with no specific treatment. Examining the spatial autocorrelation and variability of dengue prevalence throughout Bangladesh's 64 districts was the focus of this study.

**Methods:**

The spatial autocorrelation is evaluated with the help of Moran I and Geary C. Local Moran I was used to detect hotspots and cold spots, whereas local Getis Ord *G* was used to identify only spatial hotspots. The spatial heterogeneity has been detected using various conventional and spatial models, including the Poisson‐Gamma model, the Poisson‐Lognormal Model, the Conditional Autoregressive (CAR) model, the Convolution model, and the BYM2 model, respectively. These models are implemented using Gibbs sampling and other Bayesian hierarchical approaches to analyze the posterior distribution effectively, enabling inference within a Bayesian context.

**Results:**

The study's findings show that Moran Iand Geary Canalysis provides a substantial clustering pattern of positive spatial autocorrelation of dengue fever (DF) rates between surrounding districts at a 90% confidence interval. The Local Indicators of Spatial Autocorrelation cluster mapped spatial clusters and outliers based on prevalence rates, while the local Getis‐Ord *G* displayed a thorough breakdown of high or low rates, omitting outliers. Although Chattogram had the most dengue cases (15,752), Khulna district had a higher prevalence rate (133.636) than Chattogram (104.796). The BYM2 model, determined to be well‐fitted based on the lowest Deviance Information Criterion value (527.340), explains a significant association between spatial heterogeneity and prevalence rates.

**Conclusion:**

This research pinpoints the district with the highest prevalence rate for dengue and the neighboring districts that also have high risk, allowing government agencies and communities to take the necessary precautions to mollify the risk effect of DF.

## 1 INTRODUCTION

The mosquito‐borne dengue fever (DF) is the most quickly spreading disease worldwide.^1^ During an epidemic in 1870, the disease was known as “Denga” in Zanzibar, where the name “Dengue” first appeared.^2^ Classic DF, dengue hemorrhagic fever, and dengue shock syndrome are all caused by dengue virus (DENV), which is carried by day‐biting Aedes mosquitoes, primarily *Aedes aegypti* and *Aedes albopictus*.^3^ About 2.5 billion people reside in dengue‐endemic nations, with an annual estimated 50 million new dengue cases reported.^4^ As an example of a disease that could constitute a public health emergency of international concern with implications for health security due to disruption and rapid epidemic spread beyond national borders, DF is mentioned in World Health Assembly resolution WHA58.3 from 2005, which discusses the revision of the International Health Regulations (IHR).^5^, ^6^

More than 1.8 billion people (70% of the global population at risk) live in the WHO's Southeast Asia Region and Western Pacific Region, which are also responsible for more than 75% of the global sickness burden attributable to DF.^4^ Since 2000, both the number of people infected and the area affected by the dengue epidemic have increased. In 1964, DF was first recorded in eight countries: Bangladesh, India, Indonesia, Maldives, Myanmar, Sri Lanka, Thailand, and Timor‐Leste. Bhutan announced its first DF case in 2004. Nepal first reported locally‐transmitted dengue cases in November 2006. Only in the Democratic People's Republic of Korea have there been no reports of locally transmitted DENV.

In the late summer of 1964, a “Dacca Fever” outbreak in the capital city of Bangladesh (now Dhaka) led to the discovery of the country's first dengue disease.^7^ The country experienced intermittent dengue infections and brief outbreaks between 1964 and 1999, but they weren't formally documented.^8^ Since the disease first became widespread in 2000, when there were 5551 cases and 93 fatalities, it has become a serious hazard to the public's health throughout three large cities (Dhaka, Chittagong, and Khulna) and 17 towns, with a case fatality rate of 1.7%.^9^ The Ministry of Health and Family Welfare formed the CDC as a separate unit inside the DGHS's Disease Control Division in 2000, following a severe dengue outbreak in Dhaka city that year.^10^ However, a greater outbreak (6132 cases) with a case fatality rate of 1.0% was reported in 2002, after a decrease in both cases and deaths in 2001.^11^, ^12^, ^13^ As of December 2014, the Directorate General of Health Services (DGHS) had received reports of over 28,000 illnesses and 242 deaths since January 2000.^14^ An increase in dengue outbreaks from 2769 in 2017 to 10,148 in 2018 has caused increased public concern.^15^ After notifying the country of a dengue outbreak in August of 2019, the number of reported cases has dropped from 430,282 in 2019 to 59,675 in 2020.^15^ DF is a serious problem in Bangladesh, with 28,429 confirmed cases in 2021 and more cases being recorded every day.

Data science is playing an increasingly crucial role in identifying and implementing solutions to social and economic problems because of the exponential growth in the available data and the continuous improvements in information technology.^16^ Problems with crop harvesting, identifying disease trends, commercial data mining, and e‐commerce fraud are just some examples of the many areas where data science methods have been applied.^16^, ^17^, ^18^ The deadly global DF outbreak that began in 2019 has also increased the usage of data science in the healthcare industry.^19^ Researchers have utilized a variety of data science approaches, including retrospective and prospective analyses, to try to make sense of the global spread of the DENV.^20^, ^21^, ^22^ Epidemiology can be thought of as the study of disease occurrence and transmission to identify risk factors.

Box‐Jenkins was used in a study to develop an ARIMA model, which stands for autoregressive integrated moving average in^23^ to anticipate changes in the prevalence of dengue. Dengue hemorrhagic datasets from several Mexican states and union territories were categorized using cluster analysis.^24^ The purpose of this study was to make government policy and monitoring processes better. While standard statistical models have been used to evaluate demographic factors relevant to dengue transmission in Bangladesh,^25^ no studies have examined the geographical dependence of dengue cases throughout Bangladesh's 64 districts. Most established statistical methods assume initially that the data are uncorrelated. Traditional approaches are ineffective or irrelevant because they rely on assumptions of independence and homogeneity (stationarity), both of which are violated by cluster patterns.

Disease distributions are mapped using parameter estimates derived from both observational data and prior assumptions using Bayesian inference.^26^, ^27^ To create a dengue risk map, spatial and spatiotemporal modeling techniques are quite helpful.^27^, ^28^ Overdispersion and spatial correlation in the data are typically modeled using hierarchical Bayesian approaches. Random effect models, such as the Poisson‐Lognormal (PLN) and Poisson‐Gamma distributions, are two common approaches to this issue.^28^ Data overdispersion due to Poisson errors, also known as “spatially uncorrelated heterogeneity” in disease mapping, is accounted for by these two models.^29^ Overdispersion in Poisson data can arise due to spatially unstructured covariates, excessive zero counts, or counts that deviate significantly from the mean.^29^ To address these issues, two proposed models assume a gamma or lognormal distribution of random effects. The Conditional Autoregressive (CAR) model explores the spatial relationships between data and often performs better than models only correcting for unstructured heterogeneityusing weighting schemes.^29^ Both unstructured and structured random effects coexist in Convolution (COV) models.^30^, ^31^ The combined model accounts for both overdispersion and clustering through the use of gamma and normal random effects.

This study presents the results of our efforts to answer the following research topics through primary research. Does the clustering of the prevalence rate of a dengue outbreak suggest anything about the influence of the geographic features? How then do we quantify the data's geographical dependence and spatial heterogeneity? Furthermore, how can we include this in the statistical models? Do governments in a cluster look to the policies of their neighboring districts for guidance, or do they act independently? In Section 2, detailed explanations of methods and materials, including the Bayesian statistical models and sources of data, are presented. Using spatial autocorrelation and results from a suitable model, the primary contributors to the elevated prevalence rate of dengue in Bangladesh are identified in Section 3. Finally, Section 4 emphasized how the study's findings may provide government authorities with actionable information to lower the occurrence of dengue.

## 2 MATERIALS AND METHODOLOGY

### 2.1 Spatial data sources

**Figure 1:**
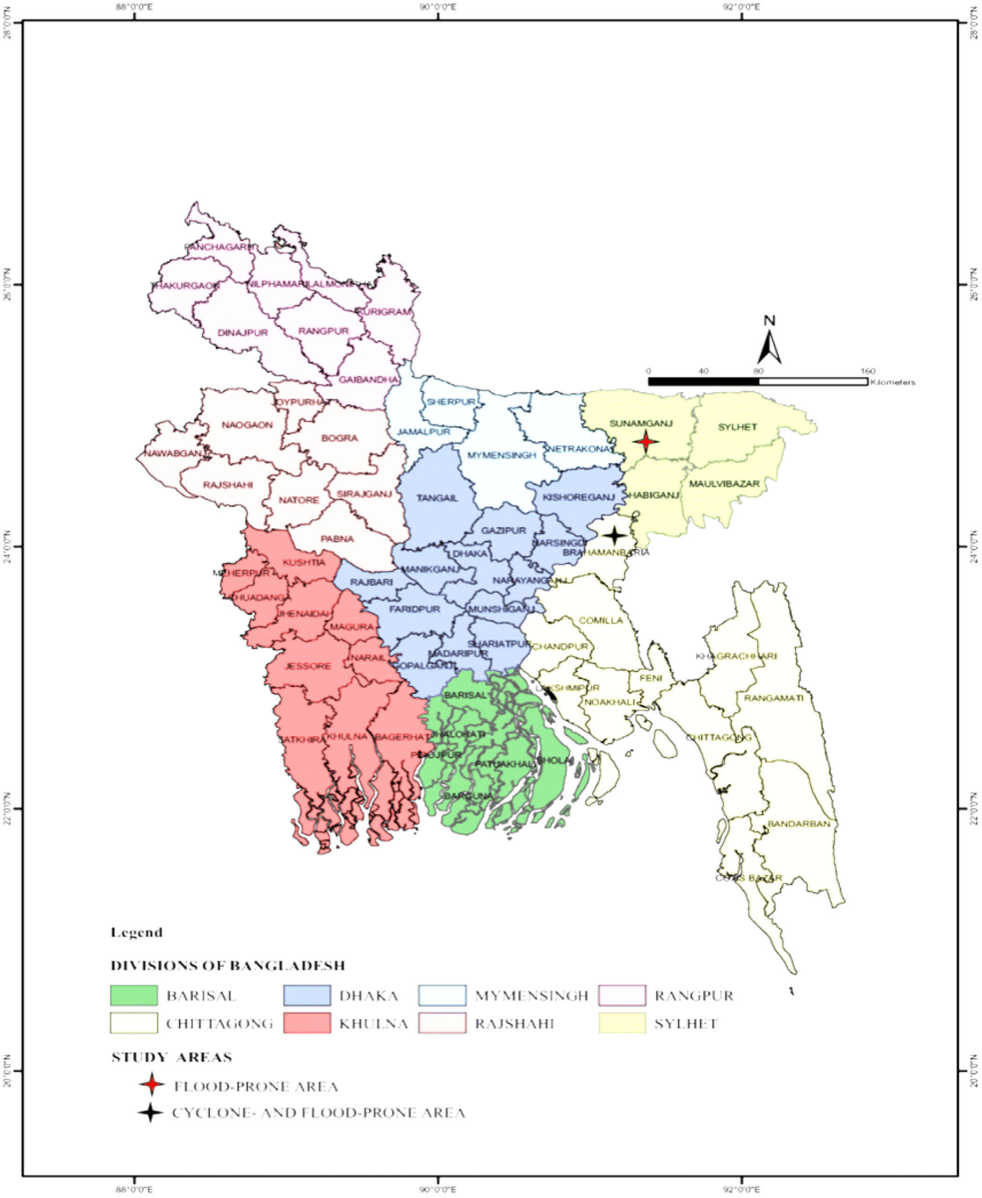
Study area of this study (district‐wise map of Bangladesh).^32^

The study area of this research is depicted in Figure 1. The data used in this article was obtained from the DGHS website (https://dghs.gov.bd/), which is publicly available. Data on the total number of afflicted patients were collected from this source beginning on January 01, 2022, and continuing through December 31, 2022. It is important to note that any personal information of individuals was not included in the data set. The other two variables, that is, the population of each district and the annual growth rate of the population by district, were taken from the census conducted by the Bangladesh Bureau of Statistics (BBS) (https://tinyurl.com/3dcspsfp) in 2011. The anticipated population of all 64 districts in 2022 was collected from (https://tinyurl.com/3j4ff8r4). Both the data sources are publicly available too. By using the geometric model, the predicted population growth rate is determined for 2022. Here is the formula for determining the rate of expansion:

$$r=\sqrt[{n}]{\frac{P}{P_{0}}}-1$$

where $P_{0}$ is the 2011 population, Pis the anticipated 2022 population, n determines the time interval between two successive censuses, and r determine the annual growth rate for 2022. According to this study, the prevalence rate formula stands for:

$$\text{Prevalence}\;\text{rate}=\frac{\mathrm{No}.\mathrm{of}\;\mathrm{Dengue}\;\mathrm{Affected}\;\mathrm{Cases}}{\mathrm{Annual}\;\mathrm{Growth}\;\mathrm{Rate}\times \mathrm{District}\;\mathrm{Population}}\times 100,000$$

The burden on the health and social care system at any given time can only be understood through a district‐by‐district prevalence rate evaluation; hence this must be carried out. After that, an estimated number of impacted people is computed per 100,000 in each district.

### 2.2 Distribution of response variable

In terms of probability distributions, the Poisson distribution is discrete. To put it another way, the variable's value can only be a whole number, such as 0, 1, 2, 3, and so forth. Neither a decimal nor a fraction will do.^33^ Below is a representation of the probability mass function (pmf):

$$\text{Pr}\left(Y=y\right)=\frac{e^{-\lambda }\lambda^{y}}{y!};\;y=0,1,2,\ldots ,n$$

here, e determines the Euler's number (*e* = 2.71828…), *λ* and *y* determine the incidence rate and the number of instances, respectively.

### 2.3 Spatial autocorrelation

According to Getis,^34^ the concept of spatial autocorrelation is one of the fundamental components of spatial analysis. It is useful for testing the hypothesis of systematic geographical variation by immediately considering the feature districts and their related values.^35^ Conventional models make the premise that observations are independent of one another; however, spatial correlation creates a divergence from that assumption.^36^ The spatial autocorrelation approach examines the geographical patterns of individual entities to determine whether they are clustered, random, or dispersed.^37^ This can be done by assessing whether or not the patterns are random, dispersed, or clustered.

### 2.4 Moran's *I* autocorrelation

A correlation coefficient known as Moran's I can be used to evaluate the degree of spatial autocorrelation in data collection. In other words, it calculates an object's degree of similarity to its immediate surroundings.^38^ The observations are not independent if there is a correlation between the items. Clustering, dispersion, and randomness are the three patterns it can identify when given a set of features and an associated characteristic. Comparing variable states over time, assessing the degree of self‐association in a given location, and determining its relationship to others are all facilitated by this tool. The values of Moran's I fluctuate between +1 and 1, where *E* (Moran's *I*) = $\frac{-1}{n-1}$.^39^ Similar to Pearson's coefficient,^40^ Moran'sI statistic has the following formula:

(1) $$I=\frac{n\sum_{i}\sum_{j}\omega_{\mathrm{ij}}Z_{i}Z_{j}}{\left(n-1\right)\sum_{i}\sum_{j}\omega_{\mathrm{ij}}}$$

In this research, n determines the total number of districts, $\omega_{\mathrm{ij}}$ is a quantification of the spatial weight of two districts i and j. The variables of interest are transformed into z‐scores, and the numerator is the sum of the products of z‐scores in neighboring districts. The first step in a spatial autocorrelation analysis is to create a spatial weight matrix that details the neighborhood structure for each site, as the weights are the row‐standardized ∑ω_*ij*_ .The term “adjacency” is used to describe neighboring administrative districts that exist close to a certain district. Administrative districts are not considered significantly influential enough to one another if they are not next to one another.^38^ A positive spatial autocorrelation will result from the presence of two positively correlated districts with large scores. If, on the other hand, two districts emerge with lower scores, then this indicates negative spatial autocorrelation.^41^ For this reason, a perfect scattering pattern is indicated by Moran's *I* value of 1. In contrast, a value of zero indicates a spatial pattern consistent with random chance, and a value of one announces a clustering pattern of perfect spatial autocorrelation. Let us consider the following hypotheses to be tested,

$H_{0}$: The data from each of the 64 districts is independent and not influenced by the data from neighboring districts, suggesting there is no autocorrelation of DF rates between neighboring sites. A random pattern exists.

$H_{1}$: The data acquired in each of the 64 districts demonstrate a nonrandom pattern or structure, as evidenced by the positive spatial autocorrelation of DF rates between neighboring districts.

However, the significance of any observed grouping depends on the p‐value. Extreme clustering occurs when z‐scores have large absolute values; nevertheless, the p value determines the statistical significance of this grouping. When both the p value and the z‐score are below the significance threshold (0.05), the null hypothesis is rejected. As a result, we can infer the existence of clustering.^42^ High p values and small z‐scores on the other hand, are consistent with accepting the null hypothesis.

### 2.5 Local Moran's *I*

Over the past few decades, many strategies for analyzing local spatial autocorrelation have emerged.^43^, ^44^, ^45^ When comparing two districts to their neighbors, one of the most used measures of how similar they are to one another is the local Moran's I.^38^ It is a more specific version of the global Moran's I statistic for assessing spatial autocorrelation. By considering the spatial interactions between each observation and its neighbors, Local Moran's I can identify local hotspots and cold spots. Individuals calculate Moran's I at the local level to identify clusters and outliers in the physical world.^46^, ^47^ Spatial statistics, according to Anselin,^46^ can identify autocorrelation of predetermined ordering in the region under investigation. To visualize the degree to which values are clustered near an observation, he creates LISA (Local Indicators of Spatial Autocorrelation).^44^ Here is the formula for determining local Moran's I:

(2) $$I_{i}=p_{i}\sum \omega_{\mathrm{ij}}p_{j}$$

Where, in this research, $p_{i}$ determines the dispersion of district *i*'s prevalence rate relative to the mean and $p_{j}$ determines the weight of district *i*'s neighbors in the statistic, adjusted for the number of neighbors.

### 2.6 Geary *C*

Geary's Cis a spatial statistical model for assessing the degree to which a data set is spatially auto‐correlated and spatially dependent.^38^ Using a new method to measure spatial patterns, it is comparable to Local Moran's Iand Moran's Imodels. As a measure of spatial autocorrelation, Geary's Ctries to determine if there is a connection between separate instances of the same event.^47^ Because the connection is multidimensional and bidirectional, understanding spatial autocorrelation is more challenging than understanding traditional autocorrelation. In this research, the functional form of Geary C, stated in,^47^ can be expressed in the following Equation (3):

(3) $$C=\frac{\left(n-1\right)\sum_{i}\sum_{j}\omega_{\mathrm{ij}}{\left(x_{i}-x_{j}\right)}^{2}}{2\omega \sum_{i}{\left(x_{i}-\overset{®}{x}\right)}^{2}}$$

where x determines the variable of interest and $\omega_{\mathrm{ij}}$ is a quantification of the spatial weight of two districts i and j. The value of Geary C ranges within [0, 2]. If the value falls within 0 ≤C<1, then it indicates positive autocorrelation among the districts. In addition, if it falls within C≥ 1, it indicates little spatial autocorrelation. On the contrary, if it falls within 1 ≤C< 2, then it indicates the presence of negative autocorrelation between the districts.

### 2.7 Local Getis Ord *G*

A variable can be changed into an independent variable by decreasing the spatial dependency in a spatially autocorrelated variable. After partitioning the original variable into two parts, filtered non spatial and residual spatial variables emerge. Determination of a suitable distance *d* is required in the transformation procedure within which neighborhood area units are dependent spatially.^48^ One process for identifying *d* includes evaluating the $G_{i}$statistic at increasing distances till no spatial autocorrelation remains.^49^ From the rise of the observation, both statistics value $G_{i}$and *d* increase, thus indicating the presence of spatial autocorrelation. The filtered observation, which is denoted by $x_{i}^{*}$ is as follows:

$x_{i^{*}}=x_{i}\left[W_{i}/\left(n-1\right)\right]/G_{i}\left(d\right)$where,$x_{i}$ is the original observation, the formula for $W_{i}$ involves summing up all the geographic connections $w_{\mathrm{ij}}$, where each link for *I* and each *j* within *d* of *i* (i≠j) is usually weighted as one. n is the number of observations, $G_{i}\left(d\right)$ refers to the spatial autocorrelation statistic developed by:^49^

$$G_{i}\left(d\right)=\sum_{j}w_{\mathrm{ij}}\left(d\right)x_{j}/\sum_{j}x_{j}i\neq j$$

### 2.8 Spatial regression models

Spatial regression is a branch of regression analysis that incorporates geographical information. The presence of spatial dependency among a set of data indicates the presence of an autoregressive process.^50^, ^51^

### 2.9 Poisson‐Gamma model

An alternative interpretation of the negative binomial distribution (Poisson model for modeling additional variation) is the Poisson‐Gamma model, a mixed model with gamma random effects for each area.^52^ This model accounts for over‐dispersion in count data, which is common in epidemiological studies like Dengue infections. Dengue case counts are assumed to be random throughout each district in this model and it follows a Poisson distribution with mean $e_{i}\theta_{i}$, that is,$y_{i}\sim \mathrm{Poisson}\left(e_{i}\theta_{i}\right)$ along with the assumption that:

$$\lambda_{i}=e_{i}\theta_{i};\;i=1,2,3,\ldots ,64$$

must be constant throughout each district. Due to the presence of unobserved heterogeneity, it is assumed that the prevalence rate parameter $\theta_{i}$ follows a gamma prior distribution with parameters a and b, which when combined with a Poisson likelihood, results in a gamma posterior. By incorporating a Gamma‐distributed random effect, it captures the variability in Dengue cases that cannot be explained solely by the mean incidence rate. This model is particularly useful for understanding the heterogeneity in Dengue infection rates across different spatial units, such as neighborhoods or regions. It can be defined as:

$$\theta_{i}\sim \mathrm{Gamma}\left(a+y_{i},b+e_{i}\right)$$

According to the Poisson‐Gamma model, the observations are presumed to be independent. When spatial data are correlated, it does not consider the spatial correlation between risk in surrounding districts and does not allow for straightforward adjustment for spatial factors.^39^ Models such as CAR, PLN, COV, and BYM2 were therefore considered.

### 2.10 PLN model

The dengue incidence in the districts of Bangladesh is a mixture of low and high. Taking into account this situation of this overdispersion we also incorporated the PLN model here. So, even in areas where Dengue cases are rare or frequent, this model can understand the differences in infection rates among those places. It's like having a magnifying glass to see the small details. The PLN model is an alternative to the Poisson‐Gamma model. The prevalence rate, denoted by $\theta_{i}$, is related to a linear predictor with a random effects component, $v_{i}$, that follows a normal distribution.^52^ It can be expressed in terms of the log‐normal model which possesses the following formula:

$$y_{i}\sim \mathrm{Poisson}\left(e_{i}\theta_{i}\right)$$

where

$$\log\left(\theta_{i}\right)=\alpha +v_{i};i=1,2,3,\ldots ,64$$

where $v_{i\;}\sim N(0,\sigma_{v}^{2})$ generates the district‐specific random effects and α determines the total level of the prevalence rate, which captures the additional Poisson variability in the log‐ prevalence rate of DF in the region i. In the Poisson‐Gamma model, $\theta_{i}\sim \mathrm{Gamma}\left(a,b\right)$where $e^{v_{i}}\sim \text{Lognormal}(0,\sigma_{v}^{2})$ with precision $\tau_{v}^{2}=\frac{1}{\sigma_{v}^{2}}$. Here, the variance of random effect ($\sigma_{v}^{2}$) reflects the amount of extra Poisson variation in the data.

### 2.11 CAR model

The CAR model considers the spatial dependency among neighboring districts, which is crucial in understanding the spread of Dengue infections. The CAR model incorporates a district‐specific random effect component to account for the ensuing variation by taking into account factors that vary consistently over area.^39^ The model was first presented in an empirical Bayes context by,^52^ and it was further expanded in a purely Bayesian environment by.^44^ The model can be written as:

$$y_{i}\sim \mathrm{Poisson}\left(e_{i}\theta_{i}\right)$$

where:

$$\log\left(\theta_{i}\right)=\alpha +u_{i};i=1,2,3,\ldots ,64$$

In this research, α determines the overall level of prevalence rate, and $u_{i}$represents specific random effects of each district. By introducing this spatially structured random effects, it captures the tendency for Dengue cases to cluster because of their close proximity to one another. In other words, this model employs a spatial correlation structure to calculate an area's risk level relative to its neighbors.^52^ An intrinsic CAR model implies that the linked heterogeneity terms will work deterministically under the assumption of a normal distribution where the mean and variance are weighted by the averages and variances of the neighboring areas,^53^ that is:

$$[u_{i}\left|u_{j},i\neq j,\tau_{u}^{2}\right]\sim N\left({\overset{®}{u}}_{i},\sigma_{i}^{2}\right)$$

$${\overset{®}{u}}_{i}=\frac{1}{\sum_{j}\omega_{\mathrm{ij}}}\sum_{j}u_{j}\omega_{\mathrm{ij}}\sigma_{i}^{2}=\frac{\sigma_{u}^{2}}{\sum_{j}\omega_{\mathrm{ij}}}$$

where mean ${\overset{®}{u}}_{i}$ determines the average spatial random effects of neighbors and $\sigma_{u}^{2}$ is the variance parameter with precision $\tau_{u}^{2}=\frac{1}{\sigma_{u}^{2}}$. In CAR model $\sigma_{u}^{2}$ controls the strength of spatial similarity. To implement the CAR model we use gamma prior for $\sigma_{u}^{2}$.

### 2.12 COV model

The COV model, by considering both spatial and temporal variations, allows researchers to capture the varying patterns infections in neighboring districts comprehensively. For instance, if one district experiences a sudden increase in Dengue cases, the COV model can assess how this outbreak might affect neighboring districts over time. It takes into account not only the immediate spatial impact but also how the infection spreads over subsequent periods. This is crucial in evaluating the spatial dynamics of Dengue transmission and the potential for outbreaks to spill over into neighboring areas. To account for spatial autocorrelation, convolutional models incorporate a random‐effects term, similar to the one used to account for over‐dispersion.^52^ It dissects random effects of district‐level into a correlated heterogeneity component $u_{i}$ and an uncorrelated heterogeneity component $v_{i}$, where $u_{i}$models the effects that vary in a predictable way between locations and $v_{i}$ represent effects that vary randomly across locations. Clayton and Kaldor similarly proposed this model in an empirical Bayes setting^54^ and Besag et al. developed it in a fully Bayesian implementation.^44^ It can be expressed as:

$$y_{i}\sim \text{Poisson}\left(e_{i}\theta_{i}\right)$$

$$\log\left(\theta_{i}\right)=\alpha +u_{i}+v_{i};\;i=1,2,3,\ldots ,64$$

It employs a structure of spatial correlation to calculate an area's prevalence rate with respect to its neighbors. It is believed that this follows a normal distribution.

$$[u_{i}\left|u_{j},i\neq j,\tau_{u}^{2}\right]\sim N\left({\overset{®}{u}}_{i},\sigma_{i}^{2}\right)$$

$${\overset{®}{u}}_{i}=\frac{1}{\sum_{j}\omega_{\mathrm{ij}}}\sum_{j}u_{j}\omega_{\mathrm{ij}}\sigma_{i}^{2}=\frac{\sigma_{u}^{2}}{\sum_{j}\omega_{\mathrm{ij}}}$$

### 2.13 BYM2 model

According to,^55^ two severe issues was pointed out with the COV model's prior choice, where the first issue addressed that the spatial component is not scaled. Another relates that the structured component ucannot be considered independently from the unstructured component v. Again in,^55^ the COV model has been parameterized again with precision parameter$\tau_{y}$ and mixing parameterφ, which lies between 0 and 1. To facilitate interpretation and transition priors, they also scale spatially structured effects which is known as BYM2 model. The BYM2 model adjusts for spatial dependence by incorporating a scaled spatially structured random effect, which captures the tendency for Dengue cases to cluster in proximity to each other more sophistically over COV model. This feature is essential for understanding how Dengue transmission spreads across geographical areas and identifying high‐risk clusters where targeted interventions may be needed. Hence, the BYM2 model is:

$$\log\left(\theta_{i}\right)=\alpha +x_{i}^{T}\delta +\frac{1}{\sqrt{\tau_{y}}}\left(\sqrt{1-\phi }v_{i}+\sqrt{\phi }u_{i}^{*}\right)+\log\left(E_{i}\right)$$

where $u_{i}^{*}$ resembles the scaled structured effect and $\frac{1}{\tau_{y}}$ is generalized variance. Hence, $\frac{1}{\tau_{y}}$ shows the marginal deviation from the model without random effects. The covariance matrix is hereafter defined as $\mathrm{Var}\left(\gamma |\tau_{\gamma },\phi \right)=\tau_{\gamma }^{-1}(\left(1-\phi \right)I+\phi \omega_{*}^{-1})$, with $\omega^{*}$ indicating an inversely scaled precision matrix. Using the standardized matrix, we obtained the marginal precision from v is $\frac{\left(1-\phi \right)}{\tau_{y}}$ and $\frac{\phi }{\tau_{y}}$ for $u^{*}$ with independent interpretability. The Dean model was introduced by scaling the neighborhood matrix integrating a new method termed penalized complexity (PC) priors, which assign priors to the hyperparameters.^55^ PC priors can be applied to mixing and precision parameters in the BYM2 model.

### 2.14 Deviance information criterion (DIC)

The DIC and a related measure $p_{D}$, which counts the number of most essential model parameters, are used to compare different models.^56^ In a Bayesian framework, it is especially important to understand how to define the effective number of parameters when dealing with complex models. Statistical significance is shown where, DIC difference > 5, but a DIC difference < 5 does not disprove the model with the higher DIC. Due to its reliance on Markov chain Monte Carlo (MCMC) results, DIC is vulnerable to sampling errors.^57^ Let the vector of parameters associated with y be, then use the model and prior independence to show that DIC is additive. Then 

$$\text{DIC}=\overset{®}{D}+p_{D}$$

The definition of posterior deviance is as follows, where D determines the posterior expected value of the deviance function:

$$p_{D}=\overset{®}{D}-D\left(\theta \right)$$

$$\overset{®}{\theta }=E\left[\theta |y\right]$$

$$\overset{®}{D}=\;E\left[D\left(\theta \right)|y\right]$$

and the Bayesian deviation, respectively.

(4) D├(θ┤)=−2ln⁡{├{f├(yⓜ|θ┤)┤}}+2lnf(y)

Under priors and independent models, it is found $f\left(y|\theta \right)=\prod_{k=1}^{k}f\left(y_{k}|\theta_{k}\right)$ and $f\left(y\right)=\prod_{k=1}^{k}f\left(y_{k}\right)$. The multiplicative conditional and marginal distributions of y contribute additively to Equation 4 in Bayesian deviation, resulting in the extreme value of y $DIC=\prod_{k=1}^{k}DIC_{k}$.^53^ When D is small, the model fits the data quite well. If the $p_{D}$ difference between the two models was small, then the simpler model was adopted.^52^

### 2.15 Data analysis

**Figure 2:**
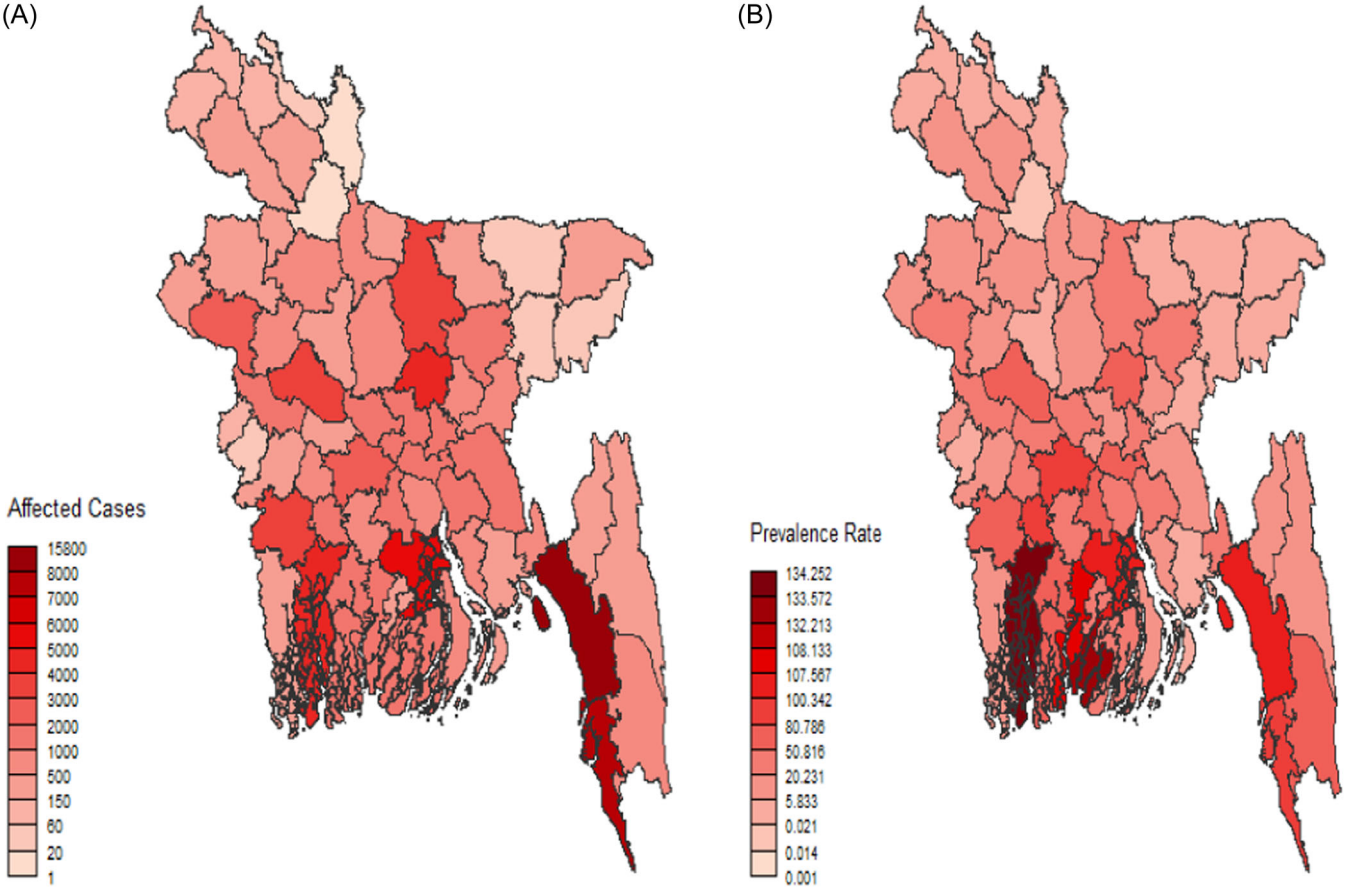
(A) District‐wise dengue affected cases; and (B) District‐wise dengue prevalence rate.

In 2022, Chattogram district had the highest number of DF cases, with a total of 15,752, making it a notable hotspot in Figure 2. Chattogram, the second‐largest city in Bangladesh after Dhaka, located in south‐eastern part of Bangladesh map, is seeing significant population expansion due to its prosperous ports, economic prospects, robust infrastructure, educational institutions, and modern amenities. Cox's Bazar and Barishalwere identified as the most affected districts in the country. On the other hand, the Gaibandha and Kurigram districts have a lower number of DF cases. Less densely populated regions, such as the hilly Bandarban, had lower reported viral cases, emphasizing the relationship between population density and disease transmission. Most cases of DF are found in tropical and subtropical locations, particularly in Southeast Asia, the western Pacific islands, Latin America, and Africa. As the primary vector for the transmission of DF, *A. aegypti* ^58^ prefers habitats with temperatures ranging from 26°C to 30°C and relative humidity levels between 70 and 80 percent. These conditions provide perfect breeding grounds^59^ and sufficient food supplies^60^ for the population to grow.

The frequency of DF varies across districts in Bangladesh, with Khulna district in the south‐west having the highest prevalence rate despite its smaller population compared to Chattogram (see Figure 2). The variation is because there are more affected individuals in Chattogram. Khulna has a higher calculated prevalence rate than Chattogram, despite its smaller population, due to its potential impact on its larger population. DF typically peaks annually from July to November, following the rainy season, with varying degrees of severity across regions. Gaibandha, located in the northern region, has the lowest rates, with Barguna and Pirojpur in the southwest following closely behind in second and third place in frequency. Barguna, with its smaller population and slower growth rate, has a more significant impact than Cox's Bazar. Pirojpur has a higher dengue prevalence rate than Barishal, despite Barishal having a lower rate due to its larger population.

**Figure 3:**
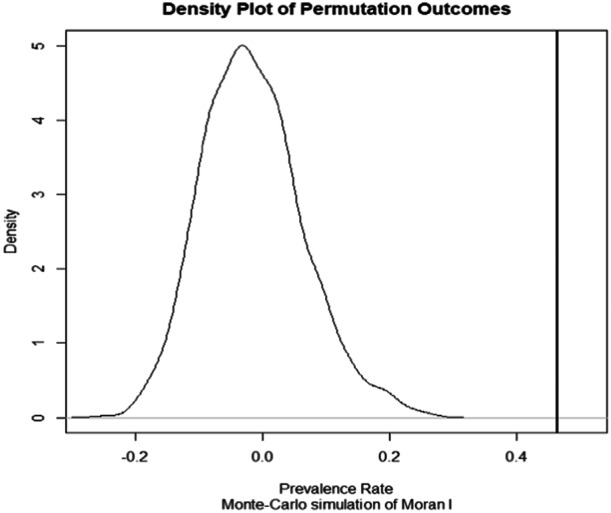
Density plot of global Moran's *I*. LISA, Local Indicators of Spatial Autocorrelation.

Table 1 shows the prevalence rate assessment of DF using the Moran's I statistic, which resulted in a positive value of 0.464, suggesting spatial autocorrelation of DF rates among districts. According to the findings of the statistical study, the null hypothesis is not supported because the p value is below 0.005 and the z‐value is above 5.939. The rejection is supported by a Monte Carlo simulation of 599 global Moran's I data points, all with p values below 0.005. The Geary Cstatistic confirms spatial clustering among districts, which values at 0.587 and falls within the range of positive autocorrelation (0−1). Clusters in the prevalence rate of contracting the DENV are found using both spatial autocorrelation approaches. The outcomes of Monte Carlo simulations are depicted graphically in Figure 3 using a density plot. This plot is skewed to the right, which supports the presence of positive spatial autocorrelation.

**Table 1:** Descriptive statistic of Moran's *I* and Geary *C* calculated under randomization.

Summary statistics	Moran's *I*	Geary *C*
Statistic	0.464	0.587
Expectation	−0.016	1.000
Variance	0.007	0.008
Standard deviation	5.824	4.534
*p* value	<0.005	<0.005
*z* value	5.937	4.679

**Figure 4:**
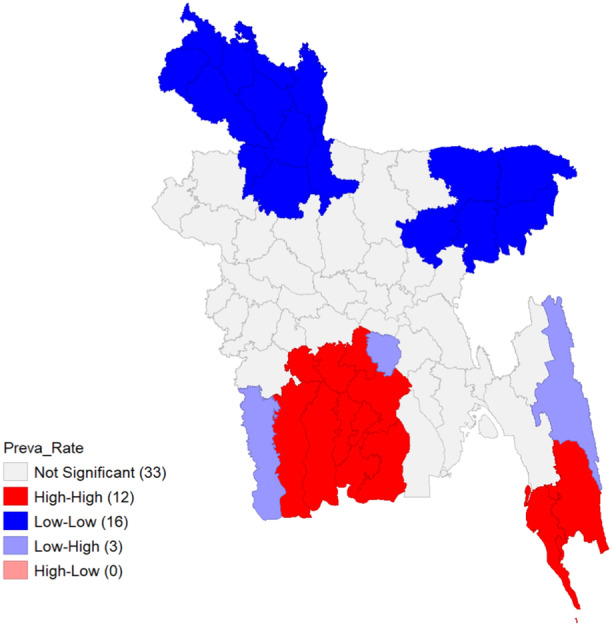
LISA cluster map for prevalence rate. LISA, Local Indicators of Spatial Autocorrelation.

Due to the influence of spatial lag and the spatial weights of the districts that are close to one another, the LISA cluster map displays the most important districts with weighted spatial homogeneity at a 90% confidence interval (see Figure 4). This widely used choropleth map categorizes locations having a high value of the local Moran's I statistic from Equation 2 according to the nature of the spatial correlation between them. The spatial clusters with high‐high intensity are depicted in red, while those with low‐low intensity are shown in blue. Low‐High spatial outliers are depicted in light blue, whereas High‐Low ones are depicted in light red. The map in Figure 4 illustrates that several districts in Bangladesh‐ Bandarban, Cox's Bazar, Narail, Bagerhat, Khulna, Pirojpur, Jhalokathi, Barguna, Barishal, Patuakhali, Madaripur, Gopalganj—are part of a significant cluster with a high−high intensity of dengue rate. The article highlights the significant occurrence of DENV in these districts and the prevalence rate of other vector‐borne diseases, suggesting that the surrounding areas pose a considerable risk. Conversely, Panchagarh, Thakurgaon, Dinajpur, Nilphamari, Lalmonirhat, Kurigram, Jamalpur, Bagura, Joypurhat, Rangpur, Gaibandha, Sylhet, Sunamganj, Moulvibazar, Habiganj, and Kishoreganj are part of a significant spatial cluster with Low‐Low intensity of dengue risk. The incidence of DF in these districts is low, similar to the surrounding areas. Despite having five times the population of Khulna, Chattogram and Khulna are both considered high‐risk zones. The population of Khulna does not align with the prevalence rate. Therefore, the city's calculated prevalence rate holds more weight than Chattogram's. Furthermore, the Low‐High spatial outliers consist of the three districts in Bangladesh: Satkhira, Shariatpur, and Rangamati. In these three districts, the prevalence rate is relatively low compared to the surrounding areas.

**Figure 5:**
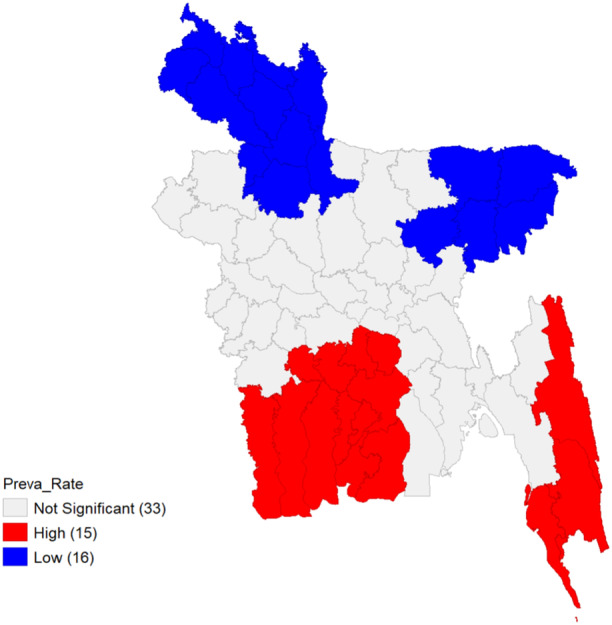
Getis Ord *Gi* map for prevalence rate.

The Local G coefficient takes an alternative approach from the Local Moran's I coefficient, which considers spatial outliers. Notably, in the Local G coefficient map, the Low‐High and High‐Low clusters found in the LISA map are reclassified as High and Low clusters, respectively. This difference stems from the underlying assumption of the $G_{i}$ index: spatial aggregation. Using concentrations of either low or high values, the $G_{i}$ index finds “hot spots” that provide low and high values for the index, respectively. Instead of capturing spatial variation in clusters like High‐High, Low‐Low, Low‐High, and High‐Low, $G_{i}$ cluster maps are made to show the geographic aggregation of High and Low zones.^49^ In Figure 5, our analysis demonstrates high prevalence rate associated with dengue in specific geographic areas. These areas encompass Bandarban, Cox's Bazar, Narail, Bagerhat, Khulna, Pirojpur, Jhalokathi, Barguna, Barishal, Patuakhali, Madaripur, Gopalganj, Satkhira, Shariatpur, and Rangamati. On the other hand, districts such as Panchagarh, Thakurgaon, Dinajpur, Nilphamari, Lalmonirhat, Kurigram, Jamalpur, Bagura, Joypurhat, Rangpur, Gaibandha, Sylhet, Sunamganj, Moulvibazar, Habiganj, and Kishoreganj exhibit clusters denoting a low prevalence rate for dengue.

**Figure 6:**
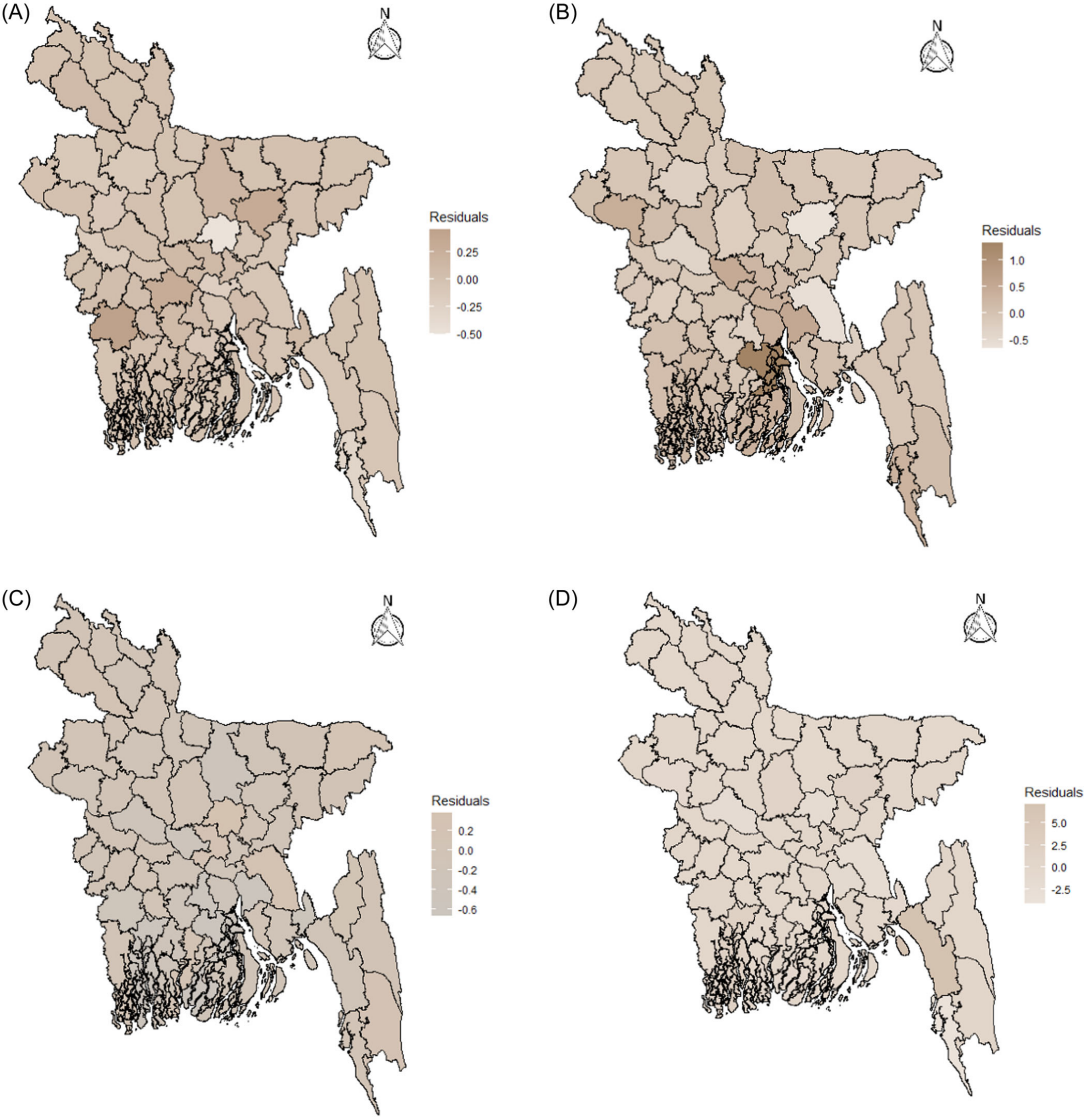
Residual plots (A) Poisson‐Lognormal model; (B) Conditional Autoregressive model; (C) Convolution model; (D) BYM2 model.

Using the likelihood and prior, the posterior distribution of Possion‐Gamma, PLN, CAR, and COV have been fitted and the posterior estimates of the distributions have been calculated is summarized in Table 2. A dependable interval indicates that the actual value is likely to fall between the lower and upper limits of the interval. The lowered $\hat{\sigma_{u}^{2}}$ value in the COV model compared to CAR indicates that the model more effectively captures the variations within each district by considering the associations among observations within each district. The $\hat{\tau_{u}^{2}}$ value for the CAR model is 18.27, indicating moderate precision and suggesting some variability in the spatial patterns. On the other hand, the value of 5766 for the same parameter in the COV model indicates that the spatial patterns or structures captured are highly consistent and well‐defined, with minimal variability. In addition, the $\hat{\tau_{v}^{2}}$ value for the COV model is high at 7882, suggesting that the nonspatial random effects are consistent and well‐defined, significantly contributing to explaining the variability in the outcome. When random factors are not considered, the marginal deviation from the model in the BYM2 model, $\frac{1}{\sqrt{\hat{\tau_{\gamma }}}}$, is 0.163, indicating that the model's efficacy in describing data patterns is enhanced by the addition of random effects. Thus, the random effects improve the model by capturing a portion of the unexplained variations in the data. The values for the DIC of five models (two nonspatial and three spatial) can be found in Table 2. A DIC discrepancy of more than 10 is a reason for favoring the model with a lower DIC value. While the DIC values of the COV model and BYM2 are close, the difference between them exceeds 10, making the lowest value of the BYM2 model acceptable. Other measuring factors, such as $\bar{D}$ and $p_{D}$ are relatively small. Hence, compared to other models, the BYM2 performed exceptionally well and explained the spatial heterogeneity of dengue infections across 64 districts. Figure 6 does not exhibit significant visual distinctions between the BYM2 model and the COV model. However, when considering DIC, the BYM2 model demonstrates a superior fit. The BYM2 model not only exhibits the lowest residual values but also successfully captures nearly all districts depicted on the thematic map because nearly all districts share the same color. There is an absence of any noticeable spatial pattern in the residual plot, which discourages additional analysis. Stationarity, the absence of autocorrelation, and a normal distribution are all observed in the data (see Appendix B).

**Table 2:** Summary statistics of Poisson‐Gamma, PLN, CAR, COV, and BYM2 models.

Posterior estimator	Poisson‐Gamma	PLN	CAR	COV	BYM2
$\hat{a}$	30.021	‐	‐	‐	‐
$\hat{b}$	0.015	‐	‐	‐	‐
Mean	1946.0	1949.0	1948.0	2049.3	2086.4
Variance	130,900.0	‐	‐	‐	‐
$\hat{\alpha }$	‐	7.574	7.574	7.625	7.643
$\hat{\tau_{v}^{2}}$	‐	20.372	‐	7882.0	‐
$\hat{\sigma_{v}^{2}}$	‐	0.051	‐	0.0001	‐
$\hat{\sigma_{u}^{2}}$	‐	‐	0.057	0.0002	‐
$\hat{\tau_{u}^{2}}$	‐	‐	18.27	5766.0	‐
$\frac{1}{\sqrt{\hat{\tau_{\gamma }}}}$	‐	‐	‐	‐	0.163
$\hat{\phi }$	‐	‐	‐	‐	0.315
95% Credible Interval	(1822.0, 2082.0)	(1837.0, 2062.0)	(1867.0, 1976.0)	(2030.0, 2087.0)	(2066.0, 2104.0)
$\overset{®}{D}$	583.901	583.244	583.296	537.210	**526.222**
$\hat{D}$	524.977	524.721	524.700	524.660	**525.105**
$p_{D}$	59.567	59.180	58.596	12.551	**10.465**
DIC	643.811	643.081	641.893	549.761	**527.340**

*Note*: Bold values are used to highlight the best model.

Abbreviations: CAR, Conditional Autoregressive; COV, Convolution; PLN, Poisson‐Lognormal.

## 3 DISCUSSION AND CONCLUSIONS

This study aims to investigate significant variations in the spatial distribution of dengue cases among 64 districts. Analyzed using Moran Iand Geary C, the district‐level spatial autocorrelation reveals insights into districts' embeddedness and spatial dependency, highlighting a significant clustering pattern among them. The LISA cluster map illustrates the districts and the neighborhoods around them that are part of spatial clusters and outliers. Excluding spatial outliers, the local Getis‐Ord G method demonstrates the districts with lower (higher) prevalence rates and their neighboring districts, providing a comprehensive view of dengue prevalence rates. Five different models were employed to examine the heterogeneity of the spatial data, revealing variations in the fitted models based on the context. After examining the statistical significance of the posterior distributions and considering the model selection criterion, it was determined that the BYM2 model is the most suitable for capturing the spatial heterogeneities in this study.

The results of this study highlight an increased risk in densely populated districts such as Chattogram, which has the highest number of cases identified. Factors such as overpopulation, rapid spread, lack of security, and a lack of preventative measures are assumed to increase the level of risk. A decreased prevalence rate will inevitably lead to fewer cases since the number of infected instances is directly proportional to the prevalence rate. Khulna has the highest prevalence rate compared to other cities in Bangladesh. Due to the combination of heavy rains, waterlogging, flooding, increased temperatures, and unpredictable changes in the district's usual seasons, the conditions in Khulna have become conducive for a dengue outbreak, worsening the situation.^61^ Evidence indicates that Barguna and Pirojpur in southwest Bangladesh pose a notable risk, though less severe than Khulna in the same geographical area. The government should share information and ensure that protective gear is readily available. Preventative measures should be within the financial reach of the general population. Reducing the mosquito population is crucial in combating diseases transmitted by mosquitoes. These measures mainly aim to get rid of places where adult mosquitoes and their larvae can breed.^62^ Despite its constraints, the first licensed vaccine for dengue, Dengvaxia (Sanofi), received approval from regulators in multiple countries.^63^ One potential solution involves the creation of a vaccine.^58^ DENVax and TV003/TV005 are two additional live‐attenuated vaccination options, but their costs make them unaffordable for most individuals. The total expense for the suggested three doses of Dengvaxia in Indonesia is around US $207.^63^ If the cost of existing mosquito repellent creams rises tenfold, we can anticipate an even more significant increase in the price of Dengvaxia. The next potential outbreak caused by mosquitoes remains unidentified. Instead of focusing on dengue or chikungunya, our efforts should be directed toward minimizing the chances of mosquito bites.^64^

Dealing with the *A. aegypti* and *A. albopictus* mosquitoes can be quite challenging due to their ability to breed in various water sources, requiring urgent attention in all regions of Bangladesh. Utilizing aerial pesticides as a proactive approach can help decrease the number of adult mosquitoes. Using residual pesticides indoors may have its benefits, but it may not be practical in crowded areas. For this investigation, we focused solely on one response variable and did not consider other factors apart from population density in the study location. By including more potential risk factors in the model, we can achieve more widely applicable results. In future investigations, we plan to incorporate Bayesian hierarchical spatial modeling and additional factors such as climatic variables, demographic variables, vector characteristics, the latent period of infections, breeding sites, and more. The findings of this research will have led to uncovered additional valuable insights.

## 4 LIMITATIONS

Every study has its limits, and our study is no exception. This analysis must account for the uncertainty in estimating the unobserved population sizes in 2022, which could impact the results, but it is challenging to address. Calculating it would have enhanced the accuracy of our investigation. Due to time constraints and lack of data, we could not include any risk factors. Incorporating risk factors into our analysis would enhance its robustness and precision. We aim to incorporate risk variables in future studies.

## AUTHOR CONTRIBUTIONS

The study was planned and overseen by Dr. Karim. Sarker was in charge of execution. She and Sefat‐E‐Barket analyzed the data and created a rough draft of the manuscript. Initially, Dr. Karim planned the work and Sefat‐E‐Barket gathered the information. Hasan reviewed the findings and data analysis. The paper was examined by each author.

## CONFLICT OF INTEREST STATEMENT

The authors declare no conflict of interest.

## ETHICS STATEMENT

We have conducted ourselveswithintegrity, fidelity, and honesty. We have not intentionally engaged in or participated in malicious harm to another person or animal. As we collected the data from the authorized data source of Bangladesh entitled DGHS (https://dghs.gov.bd/), we did not need any consent here. That's why collecting ethical approval is not related to our study.

## TRANSPARENCY STATEMENT

The lead author Sefat‐E‐Barket affirms that this manuscript is an honest, accurate, and transparent account of the study being reported; that no important aspects of the study have been omitted; and that any discrepancies from the study as planned (and, if relevant, registered) have been explained.

## Figures and Tables

**Figure A1:**
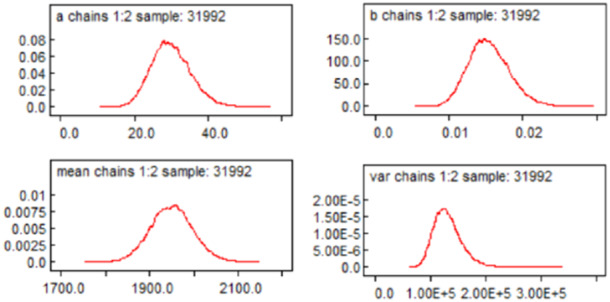
Kernel Density plots for posterior distribution obtained by Poisson‐Gamma Model.

**Figure A2:**
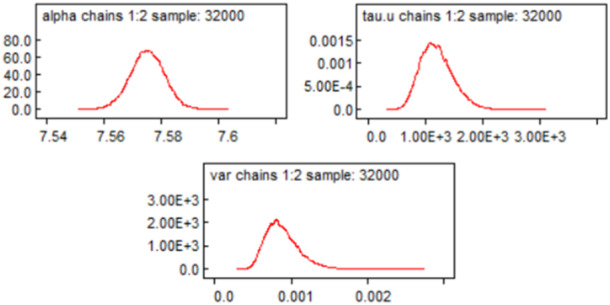
Kernel Density plots for posterior distribution obtained by Poisson‐Lognormal model.

**Figure A3:**
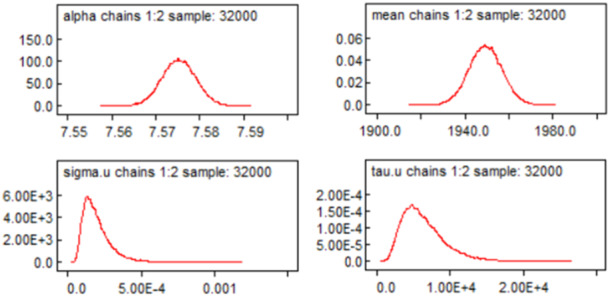
Kernel Density plots for posterior distribution obtained by CAR model. CAR, Conditional Autoregressive.

**Figure A4:**
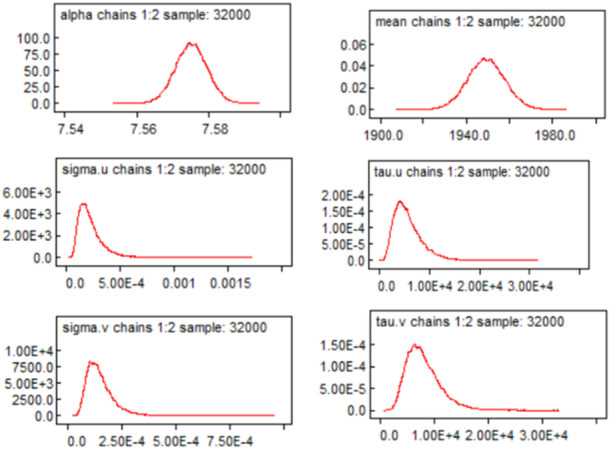
Kernel Density plots for posterior distribution obtained by COV model. COV, Convolution.

**Figure A5:**
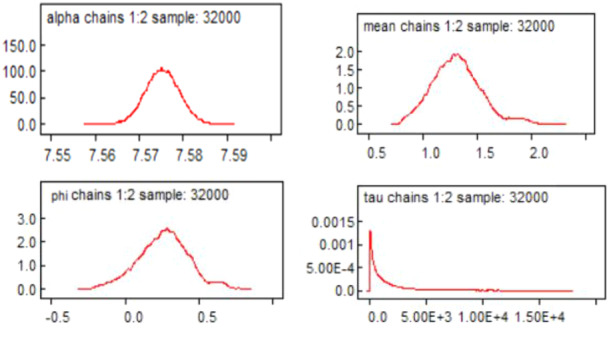
Kernel Density plots for posterior distribution obtained by BYM2 model.

**Figure A6:**
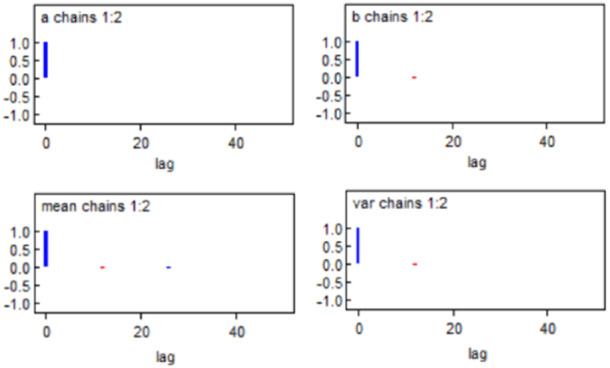
Auto‐correlation plots for posterior distribution obtained by Poisson‐Gamma Model.

**Figure A7:**
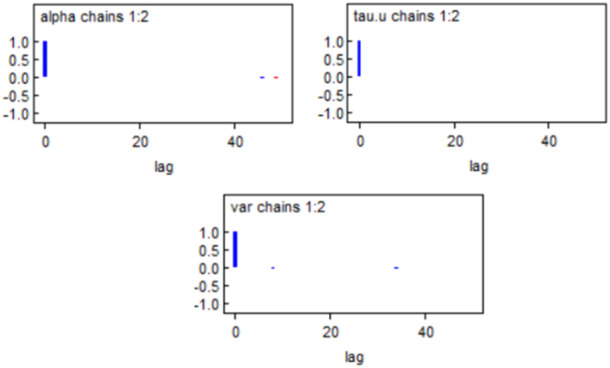
Auto‐correlation plots for posterior distribution obtained by Poisson‐Lognormal model.

**Figure A8:**
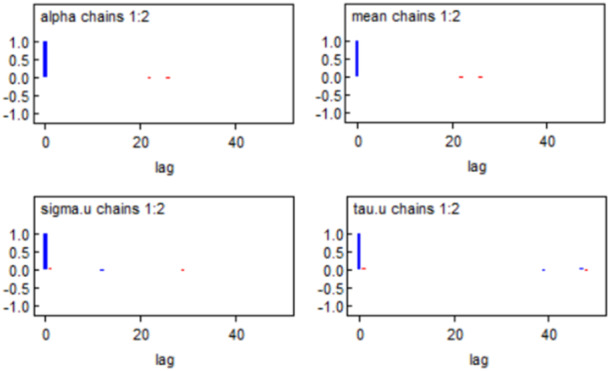
Auto‐correlation plots for posterior distribution obtained by CAR model. CAR, Conditional Autoregressive.

**Figure A9:**
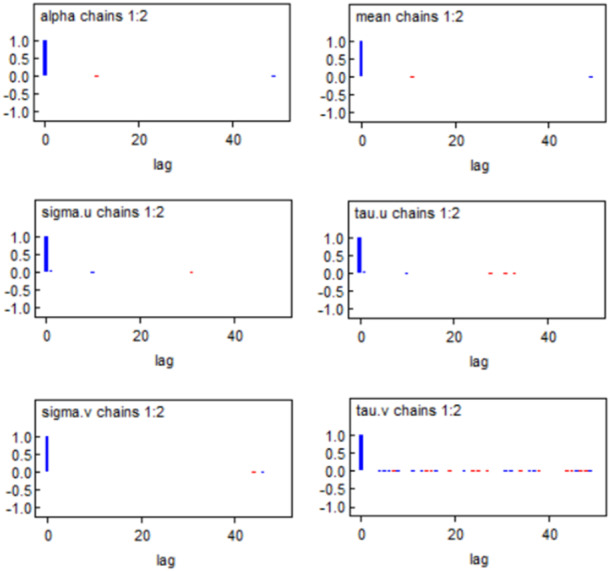
Autocorrelation plots for posterior distribution obtained by COV model. COV, Convolution.

**Figure A10:**
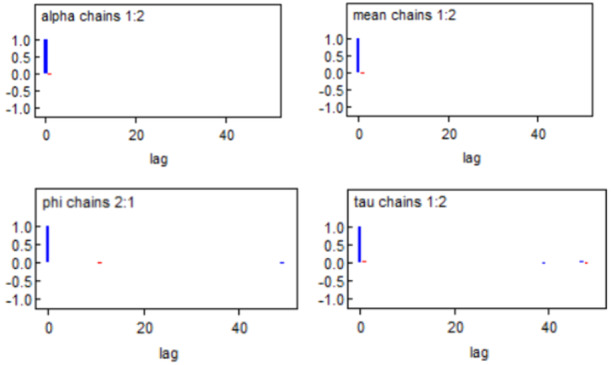
Autocorrelation plots for posterior distribution obtained by BYM2 model.

**Figure A11:**
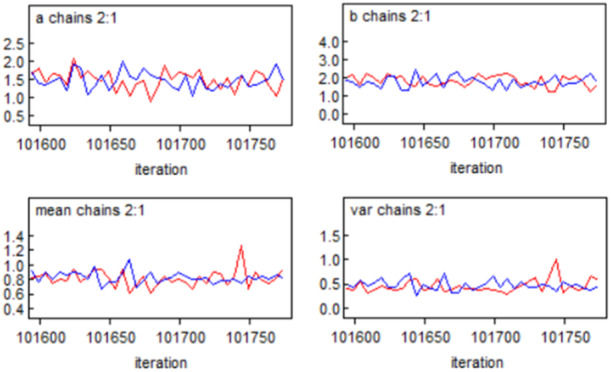
Dynamic Trace plots for posterior distribution obtained by Poisson‐Gamma Model.

**Figure A12:**
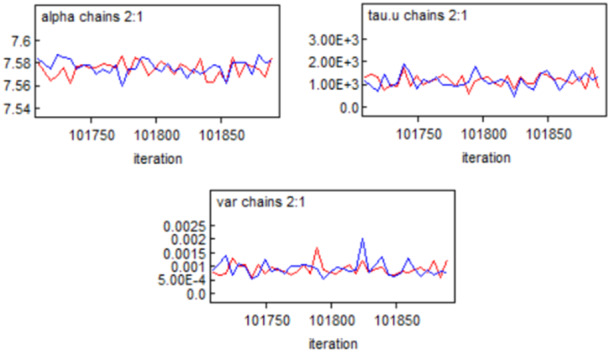
Dynamic Trace plots for posterior distribution obtained by Poisson‐Lognormal model.

**Figure A13:**
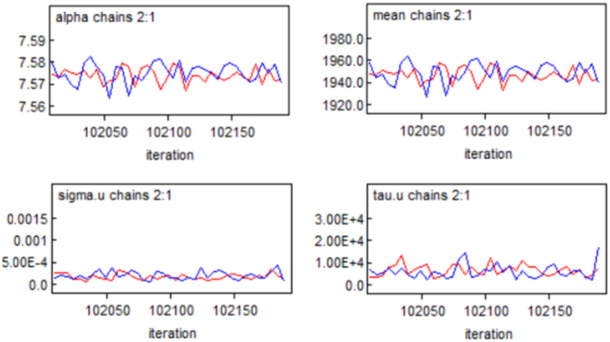
Dynamic Trace plots for posterior distribution obtained by CAR model. CAR, Conditional Autoregressive.

**Figure A14:**
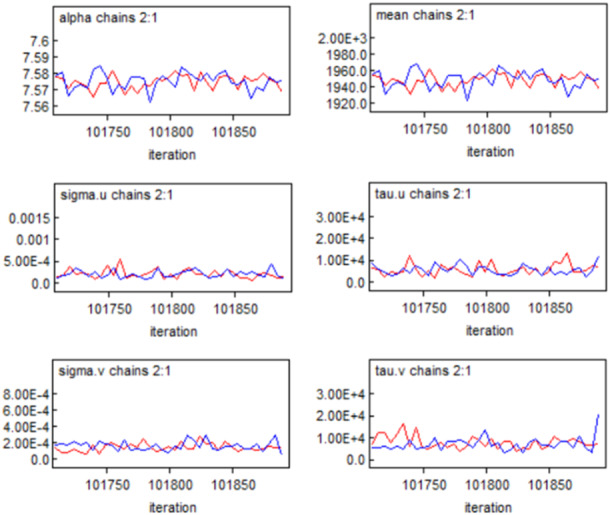
Dynamic Trace plots for posterior distribution obtained by COV model. COV, Convolution.

**Figure A15:**
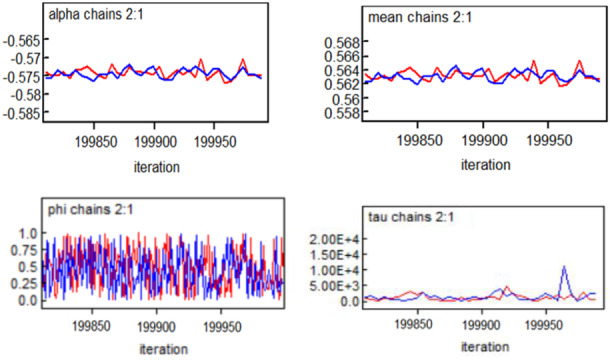
Dynamic Trace plots for posterior distribution obtained by BYM2 model.

## Data Availability

The data are available on the website, and the link is provided in Section 2. It will be provided if anyone requires this. The R code is available. It will be provided if anyone requires this.

## APPENDIX A

**Computational procedures**

To visualize the affected cases, the ***maptools*** package in RStudio was utilized. Both Moran's *I* and Geary C values along with p‐ value, z‐ value, expected values can be found in ***spdep*** packageare used by RStudio 4.2.0 to calculate spatial autocorrelation. Using a LISA cluster map, the software GeoDa version 1.20.0.10 runs 99 simulations at a 10% level of significance to identify high‐risk regions of dengue prevalence. Using the same software with the same version, a local Getis Ord G map has been produced to identify high‐ and low‐risk districts. Bayesian modeling and spatial data analysis can both be carried out with the assistance of WinBUGS. This statistical program does Bayesian analysis through the use of Markov chain Monte Carlo (MCMC). The actual value of the posterior estimators in the Bayesian hierarchical framework was calculated using two independent chains for each model, with each chain beginning with a different set of arbitrary initial values. These chains were run in parallel. The dynamic trace plots were analyzed with WinBUGS to determine whether or not the two chains were properly mixed. There were a total of 100,000 iterations, however, a burn‐in sample of the first 20,000 was deleted. Thinning values of 5 were utilized to promote convergence and lessen the impact of autocorrelation while evaluating the estimator in spatial modeling.

## APPENDIX B

The data is visually illustrated to follow a normal distribution around the mean in Figures A1, A2, A3, A4, A5 which helps to clarify the statistical significance. Nevertheless, these two characteristics, precision and variance, indicate a chi‐square distribution. Significant posterior correlations between parameters are typically linked to slow mixing, and autocorrelation plots can reveal dimensions of the posterior distribution that are mixing slowly. Figures A6, A7, A8, A9, A10 illustrate that autocorrelation diminishes rapidly and estimators blend effectively before examining any specific case. Thus, there is no indication of autocorrelation. Stationarity and excellent mixing in the trace plots are highly sought‐after qualities. The path needs to stay within the posterior distribution to be considered stationary. More specifically, each trace groups together around one incredibly stable pattern. Stationarity is illustrated in Figures A11, A12, A13, A14, A15. Second, achieving a good mix involves ensuring that no two samples within a parameter are statistically indistinguishable. As the trace moves through the posterior distribution, it meanders along various pathways. Trace plots from experiments highlight the second characteristic that helps clarify statistical significance. Both the red and blue stores utilized these options.
